# Medicine

**Published:** 1899-12

**Authors:** J. Michell Clarke


### Progress of the Medical Sciences.

### MEDICINE.

The observations of Dr. Meinhard Pfaundler on **lumbar puncture in children**[1] are interesting. From experiments on

[1] *München. med. Wchnschr.*, 1898, xlv. 1393 ; abstract in *Neurol. Centralbl.*, 1899, xviii. 507.

the pressure-conditions in the subarachnoid space, he finds that variations in the pressure have some diagnostic importance. In tuberculous meningitis not only is the pressure raised, but the pressure-curve is characteristic, as it rises during the "stage of irritation" until "the stage of pressure" and falls from the beginning of the "stage of paralysis" until death. The localisation of the exudate also influences the height of the pressure. If the pressure is found normal in a case in which there is no disturbance of the heart's action, any cerebral or meningeal affection is unlikely.

With regard to the chemical characters of the fluid removed, he finds that in the different stages of tuberculous meningitis a characteristic curve can be obtained for the amount of albumin present, and that the quantity increases with the duration of the case until death.

By means of "fractional centrifugalisation" he could nearly always demonstrate the presence of the tubercle bacillus in tuberculous meningitis, and distinguish sharply between two diplococci, which he indicates as of Weichselbaum's and Heubner's types respectively, in cases of epidemic cerebro-spinal meningitis.

As a therapeutic measure he found lumbar puncture of some palliative value in cases of high intracranial pressure, giving some relief to the patient. In 200 lumbar punctures in children he has scarcely once seen evil consequences of a serious nature.

Dr. Hermann Pfister[1] has made an exhaustive study of the **condition of the pupils and eye-reflexes in infants.** His observations were made on 300 children. He holds that it is not certainly established that the reaction of the pupil to light is always present in the newborn, and particularly in the prematurely born; it failed in one child for two weeks, and in another, a prematurely born child, for eight to ten days: in the latter, the consensual light reaction at first distinctly present, had disappeared on further testing. The diameter of the pupil increases from an average of 1.5 mm. at the end of the first month to 3.2 mm. for the seventh to the twelfth year. There is no difference in this respect between boys and girls. The magnitude of the light reaction increases from .9 mm. at the first month to 1.9 mm. at the sixth to the twelfth year, but does not show the same regularity of increase as the pupil diameter. The average amplitude is greater in girls than in boys. Diseases, unless they directly cause nervous symptoms, do not affect the light reaction.

Hippus occurred three times in the 300 children, once in a child aged two weeks as a precursor of hemorrhagic encephalitis.

Reflex closure of the eyelids was absent or uncertain in the

[1] *Arch. f. Kinderh.*, 1899, xxvi. 11; abstract in *Neurol. Centralbl.*, 1899, xviii. 172.

first month, often present in the second month, almost constant in the third, and after that never failed. Dilatation of the pupil to sensory irritation (pain), difficult to ascertain in children, was never certain in the first month, and in the second month only once positive. From the third to the sixth month it increased in frequency, so that there were only a small number of negative results. Dilatation of pupil to loud noises could only be proved in some cases, and was first obtained after the tenth week.

Drs. E. Weil and Rouvillois[1] report the case of a girl aged 10, who after a severe fright suffered from pains in the head, passing disturbance of speech, and thirty-three days after the fright from paralysis, affecting first the right side, then the whole body. The paralysis gradually passed off, but left stiffness of the right arm and leg, with a tremor resembling that of **paralysis agitans.** Other usual characteristic signs of paralysis agitans which were present were the maintenance of the trunk in a position bent forwards, flexed position of the arms, and difficulty in locomotion. The rate of the tremor corresponded to that of paralysis agitans, and the diagnosis of that disease was made. The patient's condition remained unaltered during the year that she was under observation. The authors could only find six cases of paralysis agitans in children in the literature of the disease, and these cases were not beyond dispute.

The occurrence of **nervous diseases in children** after an attack of one of the **acute specific fevers of childhood** is well known. Perhaps this connection is especially frequent in disseminated sclerosis: many cases are on record in which the disease began in this way. Dr. Haushalter[2] describes three cases of muscular atrophy in children, in one of which the disease began immediately after measles; the patient was 7 years old, and the disease took the facio-scapulo-humeral type, the first symptoms appearing at the age of 5 years. There was no other case in the family. The reaction of degeneration was obtained in the face muscles.

Prof. Allen Starr[3] reports three cases of Friedreich's disease, in two of which the symptoms of ataxy were excited by an attack of measles: all the patients were children of 13 to 16 years of age; they presented the typical group of symptoms, but in one of them the knee-jerks were exaggerated. He believes that too much stress has been laid on heredity as an etiological factor of the disease, and lays the chief blame on the infectious

[1] *Rev. mens. d. Mal. de l'Enf.*, Juin, 1899; abstract in *Neurol. Centralbl.*, 1899, xviii. 938.

[2] *Rev. de Méd.*, 1898, xviii. 445; abstract in *Neurol. Centralbl.*, 1899, xviii. 179.

[3] *J. Nerv. & Ment. Dis.*, 1898, xxv. 194.

diseases of children. He regards the pathological basis of Friedreich's disease as not confined to the lesions in the spinal cord, but, as in the case of disseminated sclerosis, comprising diffuse changes in the whole nervous system, and as evidence refers to the weakness of mind and affection of the eye-muscles present in his cases.

Dr. Dreisch[1] reports three cases in which, after an attack of measles, he observed paresis of the oculo-motor muscles and paralysis of accommodation, which, however, soon passed away completely. He compares these paralyses with the similar ones observed after diphtheria and other infectious diseases, and attributes them to the toxic products of the hypothetical measles bacillus.

Dr. L. Michaëlis[2] gives from Prof. Senator's clinic the account of a child, aged 13, who was confined to bed for eight days in February with an attack of influenza. On attempting to stand up it was found that walking was impossible from spasm of adductors of thighs, stiffness, and trembling of the legs. When examined in the following May there was spastic paresis of both legs, with increase of the knee-jerks, and clonic spasms of the muscles; there were no disturbances of sensation, or of the bladder or rectum. Recovery was perfect.

Dr. von Schultze,[3] on the **causes of acute poliomyelitis,** draws attention to the remarkable similarity of the initial symptoms of poliomyelitis, encephalitis, and labyrinthine otitis in children, and is of opinion that first of all a special form of acute meningitis occurs, which passes on either to the anterior spinal arteries or to certain parts of the cerebrum or to the cochlea and labyrinth. The causes of this meningitis, which can occasionally be demonstrated, are unknown. In a case of acute poliomyelitis lately observed, the author found in the fluid from a lumbar-puncture Weichselbaum's diplococcus as the sole organism present. He thinks that acute poliomyelitis can be produced by different micro-organisms, but how they get into the cerebro-spinal fluid is still doubtful. Besides infection through the nasal mucous membrane, an otitis media preceding labyrinthine otitis is most probable.

Dr. C. Thiry,[4] in a study of **general paresis in the young (under 20 years)** based on sixty-seven cases published since

[1] *München. med. Wchnschr.*, 1898, xlv. 627; abstract in *Neurol. Centralbl.*, 1899, xviii. 173.
[2] *Deutsche med. Wchnschr.*, 1899, xxv. 108; abstract in *Neurol. Centralbl.*, 1899, xviii. 507.
[3] *Deutsche med. Wchnschr.*, 1898, Nos. 23, 39; abstract in *Neurol. Centralbl.*, 1899, xviii. 175.
[4] "Thèse de Paris," 1898, *Gaz. hebd. de Méd.*, 1898, n. s. iii. 529; abstract in *J. Nerv. & Ment. Dis.*, 1899, xxvi. 196.

1877, says that in a majority of the cases a neuropathic family history is to be found. Syphilis plays an important part, its effects upon the general nutrition predisposing to degeneration of the nervous tissues. Both the cerebral and spinal symptoms closely resemble those found in the adult. The general course of the disease is so characteristic, even in children, that no mistakes need be made in diagnosis. Remissions do not occur, and the prognosis is most grave. Antisyphilitic treatment is unavailing.

Dr. Purves Stewart[1] gives a short summary of the symptomatology of **general paralysis of the insane occurring during childhood and adolescence.** He states that the age of onset varies from the ninth year onwards, the commonest being from 13 to 16. Both boys and girls are about equally liable. There is a history of inherited syphilis in 90 per cent. of cases: the ordinary objective signs of congenital syphilis in the teeth, bones, and eyes are very rarely present in these patients, the syphilitic poison seeming to expend itself on the nervous system. In a few cases a history of a fall or head injury preceding the onset of symptoms and perhaps precipitating it was given. The mental symptoms consist of a simple progressive dementia in a child of previously normal intelligence. The child becomes forgetful, dull, and apathetic, with occasional bursts of passion. Grandiose ideas are as a rule absent, mild delusions and hallucinations may exist. Physically, the general development is arrested; the genital organs remain infantile in type; the catamenia do not come on or cease. Fits or "congestive attacks" are common, and may be the first symptom. These may be typical epileptic fits, or attacks of "general trembling," or of speechlessness, or of loss of consciousness without convulsions. Optic atrophy is not uncommon; the pupil-symptoms resemble those found in adults. Fibrillary tremors of the face, lips, and tongue are usually present when the disease is well established; the speech becomes typically slurring and slovenly, and the handwriting resembles that of the adult general paralytic. General analgesia has been found in a number of cases. Motor symptoms and the knee-jerks vary as in adult cases. Unsteadiness of the upper extremities on voluntary movement may resemble the "intention tremor" of disseminated sclerosis. The superficial reflexes remain normal, and the sphincters are unaffected, though the latter may become uncontrolled with the progressive advance of mental dulness. The duration of the disease has varied from six months to eight and a half years. The morbid anatomy does not differ essentially from that of the adult variety of the disease.

The recognition that a form of **dementia closely resembling general paresis** may occur in the young is becoming generally admitted. Drs. Toulouse and Marchand[2] point out that many

[1] *Brain*, 1898, xxi. 39.

[2] *Soc. méd. d. Hôp.*, 1899, Juin 23; abstract in *J. Nerv. & Ment. Dis.*, 1899, xxvi. 571.

cases of paresis occurring in the very young are mistaken for cases of idiocy. In a case reported the progressive dementia began after a period of normal development. There were inequality of pupils, speech disturbances, rapid emaciation, and epileptiform attacks. *Post-mortem* cerebral atrophy, adherent meninges, proliferation of neuroglia, and other changes held to be typical of general paralysis were found.

Dr. August Hoch reports[1] two typical cases of general paralysis in two sisters, commencing at the age of 10 and 15 respectively, and Drs. van Deventer and Benders[2] two cases, one beginning at the age of 9 and the other at 11 years.

Dr. Grannelli[3] gives the case of a child who, at the age of 7, after a severe attack of scarlatina with nephritis, showed signs of beginning dementia. Her disposition changed, and she developed a general fine tremor. Later she had an epileptiform attack, and subsequently became a more or less typical case of general paralysis. The father was alcoholic and infected the mother (who developed tabes dorsalis at 44) with syphilis three or four years before the birth of the child.

J. MICHELL CLARKE.

### SURGERY.

1 *J. Nerv. & Ment. Dis.*, 1897, xxiv. 67.

2 *Psychiat. en Neurol. Bl.*, 1898, ii. 118; abstract in *Neurol. Centralbl.*, 1899, xviii. 855.

3 *Riv. quindicin. di psicol.* [etc.], 1898, ii. 213; abstract in *J. Nerv. & Ment. Dis.*, 1899, xxvi. 196.

